# Is the shock index a universal predictor in the emergency department? A cohort study

**DOI:** 10.1186/cc14228

**Published:** 2015-03-16

**Authors:** AK Kristensen, JG Holler, J Hallas, A Lassen, N Shapiro

**Affiliations:** 1 Odense University Hospital, Odense, Denmark; 2 University of Southern Denmark, Odense, Denmark; 3 Beth Israel Deaconess Medical Center and Harvard Medical School, Boston, MA, USA

## Introduction

The shock index (SI; heart rate/systolic blood pressure) is a widely reported tool to identify acutely ill patients at risk for circulatory collapse in the emergency department (ED). Because old age, diabetes, essential hypertension, and β-/Ca^2+^ channel-blockers might reduce the compensatory increase in heart rate and mask blood pressure reductions in shock or pre-shock states, we hypothesized that these factors weaken the association between SI and mortality, reducing the utility of SI to identify patients at risk.

## Methods

This was a cohort study from Odense University Hospital of all first-time visits to the ED between 1995 and 2011 (*n *= 111,019). The outcome was 30-day mortality. We examined whether age ≥65 years, diabetes, essential hypertension, and use of β-/Ca^2+^ channel-blockers modified the association between SI and mortality. The prognostic value of SI ≥1 was evaluated with diagnostic likelihood ratios.

## Results

We observed a 30-day mortality of 3%. With SI <0.7 as reference, a SI of 0.7 to 1 was associated with an adjusted OR of 2.9 (CI 2.7 to 3.2) for 30-day mortality while the adjusted OR for SI ≥1 was 10.3 (CI 9.2 to 11.5). ORs for SI ≥1 were reduced (but still significant) in patients who were older, hypertensive, or on β-/Ca^2+^ channel-blockers, whereas diabetes had no effect. The OR for SI ≥1 in patients ≥65 years was 8.2 (CI 7.2 to 9.4) compared with 18.9 (CI 15.6 to 23.0) in younger patients. β-/Ca^2+ ^channel-blocked patients had an OR of 6.4 (CI 4.9 to 8.3) versus 12.3 (CI 11.0 to 13.8) in nonusers, and the OR for hypertensive patients was 8.0 (CI 6.6 to 9.4) versus 12.9 (CI 11.1 to 14.9) in nonhypertensive patients. The OR for SI ≥1 of 9.3 (CI 6.7 to 12.9) in diabetics did not differ from the OR of 10.8 (CI 9.6 to 12.0) in nondiabetic patients. A SI of 0.7 to 1 was associated with ORs significantly greater than 1 (range: 2.2 to 3.1) with no evident differences within the subgroups. A SI measurement ≥1 was associated with lower positive likelihood ratios in patients ≥65 years, with hypertension, diabetes or using β-/Ca^2+^ channel-blockers (range 4.9 to 6.5) compared with patients not exposed to these factors (range 7.6 to 11.6).

## Conclusion

SI is independently associated with 30-day mortality in a broad population of ED patients. Old age, hypertension and β-/Ca^2+ ^channel-blockers weaken this association, but the association remains prognostic. SI ≥1 suggests substantial risk of 30-day mortality in all ED patients.

